# Assessing ChatGPT's Diagnostic Accuracy and Therapeutic Strategies in Oral Pathologies: A Cross-Sectional Study

**DOI:** 10.7759/cureus.58607

**Published:** 2024-04-19

**Authors:** Ömer Uranbey, Furkan Özbey, Ömer Kaygısız, Ferhat Ayrancı

**Affiliations:** 1 Oral and Maxillofacial Surgery, Ordu University, Ordu, TUR; 2 Oral and Maxillofacial Radiology, Ordu University, Ordu, TUR; 3 Oral and Maxillofacial Surgery, Gaziantep University, Gaziantep, TUR

**Keywords:** oral surgery, large language model, oral pathologies, chatgpt, artificial intelligence in healthcare

## Abstract

Background: The rapid adoption of artificial intelligence (AI) models in the medical field is due to their ability to collaborate with clinicians in the diagnosis and management of a wide range of conditions. This research assesses the diagnostic accuracy and therapeutic strategies of Chat Generative Pre-trained Transformer (ChatGPT) in comparison to dental professionals across 12 clinical cases.

Methodology: ChatGPT 3.5 was queried for diagnoses and management plans for 12 retrospective cases. Physicians were tasked with rating the complexity of clinical scenarios and their agreement with the ChatGPT responses using a five-point Likert scale. Comparisons were made between the complexity of the cases and the accuracy of the diagnoses and treatment plans.

Results: ChatGPT exhibited high accuracy in providing differential diagnoses and acceptable treatment plans. In a survey involving 30 attending physicians, scenarios were rated with an overall median difficulty level of 3, showing acceptable agreement with ChatGPT's differential diagnosis accuracy (overall median 4). Our study revealed lower diagnosis scores correlating with decreased treatment management scores, as demonstrated by univariate ordinal regression analysis.

Conclusions: ChatGPT's rapid processing aids healthcare by offering an objective, evidence-based approach, reducing human error and workload. However, potential biases may affect outcomes and challenge less-experienced practitioners. AI in healthcare, including ChatGPT, is still evolving, and further research is needed to understand its full potential in analyzing clinical information, establishing diagnoses, and suggesting treatments.

## Introduction

Successful treatment in oral and maxillofacial surgery requires an accurate diagnosis and an appropriate treatment plan. Another key point in healthcare is a well-functioning referral system. Clinician consultations are particularly crucial when dealing with oral pathologies associated with a risk of malignancy. Such consultations are essential for determining the timing of treatment and understanding the severity of the case. Failure to identify suspicious lesions and inappropriate consultations can have an adverse impact on patient survival [1]. Innovations in diagnostic approaches are imperative in addressing these challenges.

Oral pathologies can arise from a range of causes and present with diverse symptoms that can be diagnosed through a variety of methods. However, the diagnosis of such pathologies can be challenging due to their complexity and diversity, as well as to the limitations of conventional methods. ChatGPT possesses several advantages in the context of diagnosing oral pathologies, including leveraging the large amount of textual data available, adapting to different contexts and scenarios, providing a personalized and engaging text, and reducing the workload and time required for diagnosis [2]. It is also a useful supplement for additional information and can serve as a valuable additional reference for rare pathologies [3].

Based on machine learning (ML), ChatGPT's operation mechanism underlies the provision of these advantages. ML has had a notable impact in various domains, such as science, technology, engineering, and medicine. ML falls under the umbrella of artificial intelligence (AI), using a range of statistical, probabilistic, and optimization methods to enable computers to "learn" from previous instances and identify intricate patterns. Put simply, it constitutes a system that takes in data, identifies patterns, self-improves through data, and produces results [4]. Another form of AI, large language models (LLMs), represents a form of AI developed to imitate human language comprehension [5]. These systems employ deep learning methods, such as neural networks, and undergo training on extensive text data from diverse sources including books, articles, websites, and others. This comprehensive training equips LLMs to produce coherent and lifelike text. They analyze patterns and connections within their training data to predict words or phrases that are likely to follow in specific contexts [5]. Such high-level linguistic skills are beneficial in various natural language processing sectors, such as machine translation and text generation [6].

The purpose of this study is to highlight the remarkable identification and guidance abilities of ChatGPT, an increasingly popular component of AI, in cases of oral pathology. However, there are also concerns about integrating this easy-to-use version into the clinical routine of physicians with limited experience or practicing in very high-volume clinics. We conducted this study with medical specialists in order to assess the reliability of this tool, of potentially very great use in practice. We hypothesized that ChatGPT can offer appropriate treatment guidance and accurate diagnoses on condition that these cases are correctly and adequately identified.

The importance of comparing ChatGPT's performance with the latest developments in natural language processing, such as GPT4, is obvious. While GPT4 has been demonstrated to generate more fluent and coherent texts, the rationale behind our choice of ChatGPT stems from its accessibility, widespread usage, and established performance in various text generation tasks. Because of these advantages, OpenAI's ChatGPT 3.5 is the most widely used LLM today and is the focus of our study.

## Materials and methods

ChatGPT 3.5 was used to generate diagnoses and treatment plans for 12 retrospective cases from the archives of our Department of Oral and Maxillofacial Surgery. These retrospective case data, including patient history, complaints, lesion characteristics, and radiographic features, were prepared as scenarios by the authors of this article. The scenarios included typical clinical cases with oral pathologies identified by symptom/appearance-related keywords. The study was designed as a cross-sectional survey. The cases were presented in a standardized format, with differential diagnoses and treatment plans being requested.

The question pattern was presented in the following format: Question 1: "Provide three potential differential diagnoses for the given scenario, arranged in order of probability. Support each diagnosis with references from primary scientific literature." We then asked for the treatment plan. Question 2: "What is your treatment plan for your most likely diagnosis? Please include any consults, laboratory or radiographic studies, and treatments (medical and/or surgical) in your response."

Subsequently, attending doctors were invited to assess the complexity of the clinical scenarios and their alignment with ChatGPT's responses regarding differential diagnoses and treatment plans, using a five-point Likert scale. The difficulty scale was (1) very simple, (2) simple, (3) neutral, (4) difficult, and (5) very difficult. The agreement scale was (1) disagree, (2) partially disagree, (3) neutral, (4) partially agree, and (5) agree. The questioning was completed during August 25-27, 2023. Comparisons were drawn between the complexity of cases and agreement or disagreement with the diagnosis and treatment plans. Summary statistics were calculated, and univariate ordinal regression was performed to investigate the relationship between the difficulty of the scenarios and the quality of diagnoses and management plans.

Data analysis

Statistical analyses were performed on IBM SPSS Statistics for Windows, Version 27.0 (Released 2020; IBM Corp., Armonk, New York, United States) software. To provide a comprehensive understanding, a rich array of descriptive statistics was computed, encompassing essential measures such as the median, interquartile range, and range values for each individual item, as well as across the broader spectrum of scenarios delineating difficulty levels, diagnostic accuracy, and treatment plans. This examination aimed to unveil insights into the multifaceted aspects of the study variables. Furthermore, univariate ordinal regression analyses were applied. This analytical method allowed for an exploration of the intricate relationship between the perceived difficulty of scenarios and the accuracy of diagnostic and treatment outcomes. In documenting the results of the regression analyses, particular emphasis was placed on the presentation of odds ratios accompanied by their corresponding 95% confidence intervals. These measures not only provided a quantitative understanding of the relationships under investigation but also facilitated a nuanced interpretation of the findings. The criterion for determining statistical significance was set at p<.05.

## Results

The survey was completed by 30 attending physicians, including 12 surgery residents and 18 radiology residents. The scenarios were rated from very easy to very difficult, with a median value of 2-4 and an overall median value of 3 (Table 1 and Table 2).

**Table 1:** Summary statistics of scenario difficulty, diagnosis, and treatment agreement IQR: interquartile range; Std: standard

	Case difficulty	Diagnosis	Treatment
Mean	2.9472	3.8500	4.1278
Median	3.00	4.00	4.00
Mode	3.00	4.00	4.00
IQR range	0.25-2.00	0.00-1.25	0.00-1.00
Std. deviation	0.95876	0.87983	0.71225

**Table 2:** Difficulty of case scenarios and assessment of corresponding ChatGPT diagnosis SCC: squamous cell carcinoma; CGCG: central giant cell granuloma; AFO: ameloblastic fibro-odontoma; ACC: adenoid cystic carcinoma; OKC: odontogenic keratocyst; IQR: interquartile range; PET: positron emission tomography; EBV: Epstein-Barr virus; NK: natural killer

Scenarios	Median (IQR)	Range	Diagnosis	Median (IQR)	Range
A 62-year-old woman with a history of chronically biting her lip due to an ill-fitting prosthesis presents with a painful, ulcerated, and raised 6-cm mass on the inferior lip. She reports that the swelling has persisted for eight months and occasionally bleeds. The lesion has an irregular border, is localized to the vestibular part of the inferior lip, and is more rigid at the periphery. No palpable cervical lymph nodes are detected.	3(1)	2.00-5.00	1. SCC of the lip	4(0)	3.00-5.00
			2. Pyogenic granuloma		
			3. Fibroma		
A 67-year-old woman has a buccal expansion in the posterior crest of her edentulous mandible. The lesion appears multilocular, irregularly circumscribed, radiolucent, and destructive. Chewing triggers pain in the affected area.	3(1)	1.00-5.00	1. Ameloblastoma	4(1)	3.00-5.00
			2. CGCG		
			3. Mandibular metastasis		
A 63-year-old man was admitted to our clinic with a rapidly growing ulcerated lesion. The ulcerated pigmented lesion, which developed about one month ago, was located in the alveolar crest and buccal mucosa in the anterior region of the mandible and had a necrotic appearance. The patient also had a history of lung tumor and PET scan was performed before the patient was admitted and the patient was EBV positive.	3(1,25)	1.00-5.00	1. Metastatic SCC to the oral cavity	4(0)	2.00-5.00
			2. Primary oral SCC		
			3. Extranodal NK-/T-cell lymphoma, nasal type		
A 50-year-old woman presented to our clinic with a soft tissue ulcerative painful lesion involving the posterior palate, which had been growing slowly for two years and was asymptomatic on radiography and tomography. There was fluctuation in the lesion and palpable masses were present but aspiration test was negative.	4(1)	2.00-5.00	1. Mucocele	3(1,25)	1.00-5.00
			2. Salivary gland tumor		
			3. SCC		
A nine-year-old girl presents with a cystic lesion in the posterior mandibular region that is 3x1.5 cm in size and appears as a radiopaque-radiolucent (mixed) structure. The lesion has irregular margins and has expanded towards the bone. It is reminiscent of malformed teeth and is of a circumscribed nature.	3(1.25)	1.00-5.00	1. Dentigerous cyst with complex odontoma	4(1)	3.00-5.00
			2. AFO		
			3. Dentigerous cyst		
The patient presented with advanced gingivitis and inadequate oral hygiene. An erratic, lobulated, red/pink exophytic lesion measuring approximately 5x5 cm was observed in the maxillary anterior and premolar region, displaying bleeding and signs of malignancy.	2(1)	1.00-5.00	1. Oral SCC	4(0,25)	1.00-5.00
			2. Peripheral giant cell granuloma		
			3. Pyogenic granuloma (pregnancy tumor)		
In a 62-year-old woman, a cauliflower-like papillomatous tumor, 3x4 mm in size, in the ventral-lateral region of the tongue. There is a pedunculated lesion that is easily separated from the underlying tissue.	3(2)	1.00-5.00	1. Oral squamous papilloma	4(1)	2.00-5.00
			2. Irritation fibroma (traumatic fibroma)		
			3. Verruca vulgaris (common wart)		
A male patient, 38 years of age, presents with a slowly progressing, painless soft tissue mass at the junction of the hard and soft palate, causing bone resorption radiographically. The mass is well-defined and clearly visible.	3(1)	1.00-4.00	1. Pleomorphic adenoma (benign mixed tumor)	4(1)	2.00-5.00
			2. Schwannoma (neurilemmoma)		
			3. ACC		
A 60-year-old female patient has a white lesion resembling a macule, measuring 1x2 cm, in the vestibule of the maxillary right premolar. The lesion does not cause any symptoms and is tightly adhered to the underlying tissues within the gingival margins.	3(1)	1.00-5.00	1. Leukoplakia	4(1,25)	1.00-5.00
			2. Oral lichen planus		
			3. Fordyce granules		
A seven-year-old female patient presents with a mandibular swelling that is slowly growing and affecting both soft tissue and bone. She reported experiencing painful swelling and hypermobility of the left mandibular premolar region. A poorly defined radiolucency is evident in the lesion and bone.	3(1)	2.00-4.00	1. CGCG	4(0,25)	2.00-5.00
			2. Juvenile ossifying fibroma		
			3. OKC		
An 18-year-old male patient presented to the clinic with increasing pain in the right mandible for the past three months, with marked facial swelling and pain that was worse with activity and at night. There was no history of trauma or dental treatment in the affected area. On examination, there is a visible asymmetry of the right mandible with marked swelling extending from the right zygoma to below the lower mandibular border. Intraoral examination reveals a palpable hard mass in the right mandible, approximately 4×4 cm, palpable from the first molar to the anterior ramus border. The submandibular lymph nodes on the right are palpable, firm, and immobile. The lesion is tender for palpation. The panoramic radiograph shows a large, irregularly circumscribed, radiolucent lesion in the right mandibular body and ramus. Radiographically, the lesion has a mixed radiolucent/radiopaque appearance.	3(0,25)	1.00-5.00	1. Ameloblastic fibroma or ameloblastic fibrosarcoma	4(0)	2.00-5.00
			2. Central odontogenic fibroma		
			3. Osteosarcoma of the jaw		
A 42-year-old woman presented with a three-month history of painless pigmentation of the left upper anterior gingiva. There was no history of systemic disease or head and neck trauma. On examination, a palpable, tender, hard lymph node was found under the left mandible and a separate, painless, mobile lymph node on the right side. Intraoral examination revealed a non-tender, black pigmentation on the left maxillary gingiva extending to the adjacent teeth and palatal gingiva.	4(1)	1.00-5.00	1. Malignant melanoma of the oral cavity	3(1)	2.00-5.00
			2. SCC (oral cancer)		
			3. Lymphoma		

The range differed for each scenario, with interquartile ranges between 0.25 and 2 (Table 1). Table 2 and Table 3 show the case scenarios and corresponding ChatGPT responses. Acceptable agreement was observed for the accuracy of the differential diagnoses provided by ChatGPT, with a median range of 3-4 and an overall median of 4. The IQR for each diagnosis evaluation was small, usually 1.25 or less (Table 1 and Table 2). Although the scenario difficulty was defined as neutral level, there appears to have been a successful inclination to engage in diagnosis and treatment. In particular, high agreement was observed with the treatment plan provided by ChatGPT for the most likely diagnosis, with the great majority scoring (85.3%) at least 4.00 (Table 1, Table 3, and Table 4).

**Table 3:** Agreement on treatment of corresponding ChatGPT diagnosis IQR: interquartile range; SCC: squamous cell carcinoma; CT: computed tomography; MRI: magnetic resonance imaging; TNM: TNM Classification of Malignant Tumors; CGCG: central giant cell granuloma; NSAIDs: nonsteroidal anti-inflammatory drugs; PDT: photodynamic therapy; CBC: complete blood count

Prompt	Treatment management of the most likely diagnosis	Median (IQR)	Range
1	Consultation with an oral and maxillofacial surgeon or oncologist. A biopsy to confirm the diagnosis of SCC and the tumor's grade and stage. Imaging studies: CT or MRI may be ordered to determine the extent of tumor involvement, assess lymph node status, and identify any distant metastasis. Staging: the stage of the cancer with the TNM system. Treatment options: surgery: surgical resection/local excision with or without neck dissection. Radiation therapy in combination with surgery. Chemotherapy: as adjuvant therapy after surgery. Follow-up supportive care: manage pain, improve quality of life, and address any side effects of treatment	4(1)	2.00-5.00
2	Consultation with an oral and maxillofacial surgeon. Radiographic studies: radiographic studies, such as panoramic radiographs, CT scans, or cone-beam CT scans. Biopsy: for histopathological examination Staging and assessment: surgical intervention: enucleation and curettage, segmental resection, mandibulectomy, and reconstruction: this can involve the use of bone grafts, microvascular surgery, or prosthetic devices. Follow-up tendency to recur. Dental rehabilitation: functional and aesthetic issues, such as prosthetic dentures or dental implants	4(1)	3.00-5.00
3	Consultations with an oncologist and dermatologist. Laboratory biopsy imaging studies: CT scans, MRI, or PET for metastasis and to evaluate the involvement. Treatment: surgical intervention, removal of the primary lesion and affected lymph nodes. Systemic therapy: consult with the oncologist for immunotherapy or targeted therapy. Immunotherapy with checkpoint inhibitors like pembrolizumab or nivolumab radiation. Therapy as adjuvant treatment. Follow-up for recurrence. Supportive care: psychological support and pain management	4(0)	3.00-5.00
4	Clinical evaluation: consultation with an oral and maxillofacial surgeon or oral pathologist. Diagnostic imaging: panoramic radiograph or cone-beam CT. Surgical management: excisional biopsy or incisional biopsy of the mucocele, complete surgical excision, cryotherapy, or laser ablation. Postoperative care: NSAIDs or analgesics. Regular follow-up visits. Histopathological examination to ensure complete removal. Patient education	4(1)	2.00-5.00
5	Clinical evaluation: by an oral and maxillofacial surgeon or a pediatric dentist. Radiographic studies: panoramic X-rays or cone-beam CT, to confirm and visualize the cystic lesion and the presence of tooth-like structures (odontoma). Consultation with a pathologist for histopathological examination. Surgical excision: enucleation of lesion. Postoperative follow-up and monitor for any potential complications, such as infection or nerve injury. Rehabilitation includes reconstructive surgery or orthodontic treatment to restore proper dental function and aesthetics. Long-term follow-up for recurrence	4(1)	3.00-5.00
6	Confirm the diagnosis: biopsy and histopathological examination. Staging and evaluation: determine the stage of the SCC, which involves assessing the size of the tumor, extent of local invasion, involvement of lymph nodes, and presence of distant metastasis. Conduct imaging studies, such as CT scans or MRI, to assess the extent of the tumor. Consultation with specialists: multidisciplinary team, including oral and maxillofacial surgeons, medical oncologists, radiation oncologists, and dental professionals. Treatment modalities: surgery, radiation therapy, and chemotherapy. The choice depends on the stage, location, and extent of the tumor. Dental evaluation and care: address dental issues, such as extraction of non-restorable teeth, before initiating treatment. Maintain oral hygiene and provide preventive dental care during and after treatment. Supportive care: manage symptoms and side effects of treatment, such as pain, nausea, and xerostomia. Provide nutritional support as needed to ensure the patient's well-being. Regular follow-up: detect any recurrence. Psychological and social support	4(1)	2.00-5.00
7	Clinical evaluation: examination by an oral and maxillofacial surgeon or an oral pathologist. Histopathological examination (biopsy): rule out any malignancy. This is typically done through an incisional or excisional biopsy. Radiographic studies (if necessary): in some cases, imaging studies such as dental X-rays or cone-beam CT may be performed to assess the lesion's relationship with underlying structures or to rule out any additional lesions or abnormalities. Consultation with a pathologist. Surgical excision: if the diagnosis is confirmed as an oral squamous papilloma, the primary treatment is surgical excision. Provide postoperative care instructions, including hygiene and wound management, to the patient. Long-term follow-up	4(1)	3.00-5.00
8	Treatment plan for pleomorphic adenoma: consultation with specialists: oral and maxillofacial surgeon or otolaryngologist (ENT specialist). Imaging studies: CT scan or MRI biopsy: Typically, a fine-needle aspiration biopsy is used. Surgical excision: the primary treatment for pleomorphic adenoma is surgical excision and complete removal. The surgical approach may involve enucleation or partial resection, depending on the tumor's size and location. Pathological examination reconstruction and follow-up: pleomorphic adenomas have the potential for recurrence, so long-term follow-up is essential to monitor for any signs of regrowth	4(1)	3.00-5.00
9	Consultation with specialists: an oral and maxillofacial surgeon or an oral medicine specialist. Clinical examination: examination of the lesion, size, location, appearance, and any associated symptoms. Biopsy: consider obtaining a biopsy of the leukoplakic lesion to determine its histopathological characteristics and assess the degree of dysplasia. The biopsy helps rule out malignancy and provides critical information for treatment planning. Histopathological evaluation: send the biopsy specimen for histopathological examination to determine the presence and grade of dysplasia, which guides further management decisions. Tobacco and alcohol cessation: reduce the risk factors associated with leukoplakia. Management of risk factors: identify risk factors for leukoplakia, such as chronic irritation or ill-fitting dental appliances. Regular follow-up: schedule regular follow-up appointments to monitor the lesion's progress and assess for any changes. Patients with dysplastic leukoplakia may require more frequent monitoring. Treatment of dysplasia (if present): if dysplasia is identified in the biopsy, management may involve surgical excision of the dysplastic area, if localized, PDT or laser therapy for extensive lesions, and close monitoring and surveillance for progression to carcinoma in situ or invasive carcinoma. Patient education: the potential for malignant transformation and the importance of regular follow-up appointments. Lifestyle modification: encourage the patient to maintain good oral hygiene practices and a well-balanced diet. Referral for psychological support: consider referring the patient for psychological support if there is significant anxiety or emotional distress related to the diagnosis	4(1)	2.00-5.00
10	Consultation with specialists: refer the patient to an oral and maxillofacial surgeon or an oral surgeon with expertise in managing jaw lesions for a comprehensive evaluation. Clinical examination: conduct a thorough clinical examination to assess the size, extent, and characteristics of the CGCG. Imaging studies: panoramic radiographs and cone-beam CT scan or MRI biopsy: incisional biopsy or fine-needle aspiration biopsy to confirm the diagnosis and obtain a histopathological assessment. Histopathological examination: presence of giant cells and characteristic features of CGCG. Treatment options: the choice of treatment depends on the size, location, and aggressiveness of the CGCG. Surgical excision is the primary treatment option. In some cases, curettage (scraping) of the lesion may be sufficient for smaller, less aggressive lesions. If the lesion has caused significant bone loss, reconstruction may be necessary after excision. Long-term follow-up recovery and assessment for any signs of recurrence. CGCGs have a potential for recurrence. Patient education	4(1)	3.00-5.00
11	Consultation with a maxillofacial surgeon or oral surgeon. Radiographic studies: a cone-beam CT scan or CT scan with a focus on the mandible and the extent of the lesion is typically ordered. Biopsy: a surgical biopsy or fine-needle aspiration ameloblastic fibroma (benign) and an ameloblastic fibrosarcoma (malignant transformation). Laboratory studies: routine preoperative laboratory tests, including CBC, liver function tests, and renal function tests, may be ordered to assess the patient's overall health and suitability for surgery. Treatment options: if the lesion is confirmed as an ameloblastic fibroma (benign), the primary treatment is surgical resection. The goal is to remove the tumor while preserving as much healthy mandibular bone and dental structures as possible. In some cases, enucleation (removal of the lesion without resection of the surrounding bone) may be considered for small lesions that are well-defined. Reconstruction of the mandible may be necessary depending on the extent of bone involvement. This can involve techniques such as bone grafting. Postoperative follow-up. Orthodontic evaluation (if needed): long-term monitoring: ameloblastic fibromas, although benign, can have a recurrence rate, especially if not completely removed during surgery	4(0)	4.00-5.00
12	Consultation with a multidisciplinary team of specialists, an oral and maxillofacial surgeon, a head and neck surgeon, a medical oncologist, and a radiation oncologist. Biopsy: a biopsy of the pigmented growth on the left maxillary gingiva should be performed to confirm the diagnosis and determine the extent of the melanoma. Imaging: CT scans or MRI for the presence of metastases in nearby lymph nodes or distant organs. Surgical excision: the primary treatment for oral melanoma often involves wide surgical excision of the tumor, including surrounding tissues. The goal is to achieve negative surgical margins while preserving as much function and aesthetics as possible. Lymph node evaluation: given the presence of palpable lymph nodes, sentinel lymph node biopsy or lymph node dissection may be necessary to assess lymph node involvement. Reconstructive surgery: depending on the extent of tissue removal, reconstructive surgery may be required to restore form and function to the affected area. Adjuvant therapies: radiation therapy and immunotherapy. Immunotherapy: checkpoint inhibitors, in cases of advanced or metastatic melanoma. Regular follow-up for signs of recurrence and to manage any potential side effects of treatment. Psychosocial support	4(0,5)	2.00-5.00

**Table 4:** Summary statistics depending on Likert's scores

	Case difficulty		Diagnosis		Treatment	
Valid	Frequency	Percent	Frequency	Percent	Frequency	Percent
1	22	6.1	3	0.8	-	-
2	90	25.0	32	8.9	9	2.5
3	152	42.2	56	15.6	44	12.2
4	77	21.4	194	53.9	199	55.3
5	19	5.3	75	20.8	108	30.0
Total	360	100	360	100	360	100

**Figure 1:**
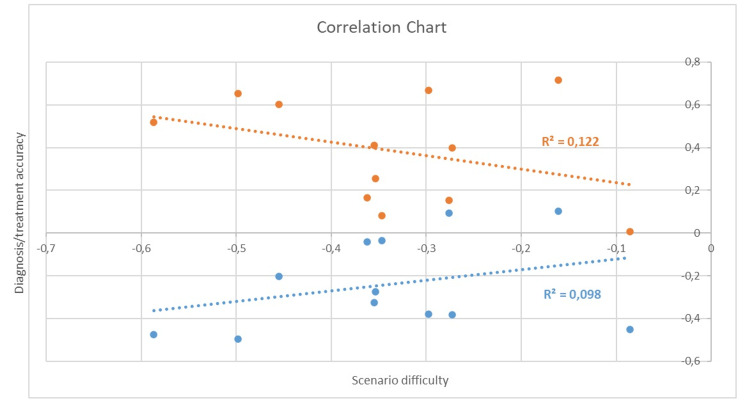
Correlation chart for diagnosis, treatment, and scenario difficulty

An association was also observed between scenario difficulty and agreement with differential diagnosis or treatment. A decrease in diagnosis scores was correlated with a greater likelihood of lower treatment management scores (Figure 1).

Orange dots show a positive correlation between diagnosis and treatment, while blue dots show a negative correlation between scenario difficulty and diagnosis/treatment accuracy.

The relationship between the difficulty of clinical scenarios and diagnostic and treatment accuracy was then examined using a univariate ordinal regression test (Table 5).

**Table 5:** Univariate ordinal regression test on each Likert score

	Dependent: diagnosis score; independent: scenario difficulty			Dependent: treatment score; independent: scenario difficulty			Dependent: treatment score; independent: diagnosis score		
Likert score	Estimate	%95 CI	P-value	Estimate	%95 CI	P-value	Estimate	%95 CI	P-value
1	0.498	(-0.666, 1.662)	0.402	0.470	(-0.721, 1.661)	0.439	-1.721	(-4.017, .575)	0.142
2	0.638	(-0.298, 1.573)	0.182	0.584	(-0.378, 1.547)	0.234	-2.077	(-2.92, -1.235)	0.000
3	0.773	(-0.129, 1.675)	0.093	0.462	(-0.465, 1.389)	0.329	-2.063	(-2.778, -1.349)	0.000
4	-0.156	(-1.098, .786)	0.745	0.309	(-0.666, 1.285)	0.534	-1.322	(-1.861, -0.783)	0.000
5	0	-	-	0	-	-	0	-	-

We used the "Agree" score (a five-point Likert-type scale) as the reference level. Table 5 presents odds ratios with their corresponding 95% confidence intervals and p-values. The relationship between independent scenario difficulty and dependent diagnosis score was examined. However, as all p-values exceeded 0.05, indicating no statistically significant effect, it was concluded that scenario difficulty did not significantly impact the diagnosis score. Similarly, in the investigation of the independent scenario difficulty and dependent treatment score relationship, no statistically significant effect was found, as all p-values also surpassed 0.05. Consequently, it was determined that scenario difficulty did not exert a significant influence on the treatment score. Conversely, when exploring the impact of the dependent treatment score on the independent diagnosis score, notable findings emerged. Specifically, for Likert scores 2, 3, and 4, p-values were less than 0.05, signifying a statistically significant effect of the diagnosis score on the treatment score for these particular scores. Thus, while scenario difficulty did not demonstrate a significant impact on diagnosis or treatment scores, a noteworthy relationship between diagnosis and treatment scores was unveiled. Overall, this significance implies that when there is less agreement with the diagnosis, there also tends to be less agreement with the treatment. The p-value for Likert score 1 was p>0.05, and this was therefore not statistically significant. The small sample size may have influenced this result.

Inclusion of another AI tool

To enhance the comprehensiveness of our study and investigate potential variations in outputs, we incorporated the use of Gemini (Google AI), another freely available AI tool. This tool employs distinct architectures and methods in contrast to ChatGPT and GPT4 and may generate diverse outputs for the same prompts. Table 6 presents the results of the review of Gemini using 12 case sets and identical question patterns (Table 6).

**Table 6:** Gemini's answers to case scenario prompts The bold-colored diagnoses are common to both LLM for the same case scenario. LLM: large language model; SCC: squamous cell carcinoma; CBC: complete blood count; CRP: C-reactive protein; EBV: Epstein-Barr virus

Prompts	Diagnosis	Treatment
A 62-year-old woman with a history of chronically biting her lip due to an ill-fitting prosthesis presents with a painful, ulcerated, and raised 6-cm mass on the inferior lip. She reports that the swelling has persisted for eight months and occasionally bleeds. The lesion has an irregular border, is localized to the vestibular part of the inferior lip, and is more rigid at the periphery. No palpable cervical lymph nodes are detected.	1. Chronic actinic cheilitis	Consults: dermatologist
	2. SCC	Treatments: non-surgical: sun protection
	3. Pyogenic granuloma	Topical medications: 5-fluorouracil, imiquimod, or diclofenac
		Surgical/destructive: cryotherapy, electrocautery, laser ablation, vermilionectomy
		Additional considerations: smoking cessation, a biopsy confirmation
A 67-year-old woman has a buccal expansion in the posterior crest of her edentulous mandible. The lesion appears multilocular, irregularly circumscribed, radiolucent, and destructive. Chewing triggers pain in the affected area.	1. Peripheral ossifying fibroma	Consults: dentist or oral surgeon
	2. SCC with bone involvement	Examination: X-rays, biopsy
	3. Mandibular torus	Treatments: surgical excision
		Additional considerations: addressing underlying causes, improved oral hygiene practices, or adjustments to dentures
		Monitoring: any signs of regrowth
A 63-year-old man was admitted to our clinic with a rapidly growing ulcerated lesion. The ulcerated pigmented lesion, which developed about one month ago, was located in the alveolar crest and buccal mucosa in the anterior region of the mandible and had a necrotic appearance. The patient also had a history of lung tumor and PET scan was performed before the patient was admitted and the patient was EBV positive.	1. SCC	Consults: oral and maxillofacial surgeon, medical oncologist, radiation oncologist
	2. Pyogenic granuloma	Laboratory and radiographic studies: biopsy imaging studies: panoramic X-ray, CT scan, PET scan
	3. Peripheral ossifying fibroma	Treatments (medical and/or surgical): wide local excision, mandibulectomy, maxillectomy
		Additional treatments: radiation therapy, chemotherapy, targeted therapy, reconstruction
		Supportive care: pain management, nutritional counseling, and speech therapy
A 50-year-old woman presented to our clinic with a soft tissue ulcerative painful lesion involving the posterior palate, which had been growing slowly for two years and was asymptomatic on radiography and tomography. There was fluctuation in the lesion and palpable masses were present but aspiration test was negative.	1. SCC	Consults: oral and maxillofacial surgeon, medical oncologist, radiation oncologist
	2. Minor salivary gland tumor	Laboratory and radiographic studies: biopsy imaging studies: panoramic X-ray, CT scan, PET scan
	3. Benign inflammatory processes	Treatments (medical and/or surgical): wide local excision, mandibulectomy, maxillectomy
		Additional treatments: radiation therapy, chemotherapy, targeted therapy, reconstruction
		Supportive care: pain management, nutritional counseling, and speech therapy
A nine-year-old girl presents with a cystic lesion in the posterior mandibular region that is 3×1.5 cm in size and appears as a radiopaque-radiolucent (mixed) structure. The lesion has irregular margins and has expanded towards the bone. It is reminiscent of malformed teeth and is of a circumscribed nature.	1. Dentigerous cyst	Consults: oral and maxillofacial surgeon
	2. Keratocystic odontogenic tumor	Laboratory and radiographic studies: X-ray or panoramic X-ray, cone-beam CT scan
	3. Calcifying odontogenic cyst	Treatments (surgical): enucleation, marsupialization
		Additional considerations: antibiotics, bone grafting
		Monitoring: for any recurrence
The patient presented with advanced gingivitis and inadequate oral hygiene. An erratic, lobulated, red/pink exophytic lesion measuring approximately 5×5 cm was observed in the maxillary anterior and premolar region, displaying bleeding and signs of malignancy.	1. SCC	Consults: oral and maxillofacial surgeon
	2. Peripheral giant cell granuloma	Laboratory and radiographic studies: X-ray or panoramic X-ray, cone-beam CT scan
	3. Pyogenic granuloma	Treatments (surgical): enucleation, marsupialization
		Additional considerations: antibiotics, bone grafting
		Monitoring: for any recurrence
In a 62-year-old woman, a cauliflower-like papillomatous tumor, 3×4 mm in size, in the ventral-lateral region of the tongue. There is a pedunculated lesion that is easily separated from the underlying tissue.	1. Squamous papilloma	Consults: dermatologist or otolaryngologist
	2. Focal epithelial hyperplasia	Laboratory and radiographic studies: biopsy, X-ray, or CT scan
	3. Verrucous carcinoma	Treatments: observation, surgical excision (scalpel, laser, or electrosurgery), cryotherapy
		Topical medications: (salicylic acid) interferon injections
		Regular follow-up for recurrence
A male patient, 38 years of age, presents with a slowly progressing, painless soft tissue mass at the junction of the hard and soft palate, causing bone resorption radiographically. The mass is well-defined and clearly visible.	1. Peripheral ossifying fibroma	Consults: oral and maxillofacial surgeon
	2. Inflammatory fibrous hyperplasia	Laboratory and radiographic studies: X-ray or CT scan, biopsy
	3. Palatal cyst	Treatments: observation, surgical excision: scalpel excision, laser surgery, or electrosurgery, enucleation
		Additional considerations: medications (NSAIDs)
		Recurrence: regular follow-up for any signs of regrowth
A 60-year-old female patient has a white lesion resembling a macule, measuring 1×2 cm, in the vestibule of the maxillary right premolar. The lesion does not cause any symptoms and is tightly adhered to the underlying tissues within the gingival margins	1. Focal fibrous hyperplasia	Consults: oral and maxillofacial surgeon or dentist
	2. Oral lichen planus	Laboratory and radiographic studies: biopsy, imaging studies (X-ray or CT scan)
	3. SCC	Treatments: observation, surgical excision (scalpel excision, laser surgery, or electrosurgery)
		Additional considerations: recurrence: monitor for any signs of regrowth
		No medications: medications are generally not used to treat
A seven-year-old female patient presents with a mandibular swelling that is slowly growing and affecting both soft tissue and bone. She reported experiencing painful swelling and hypermobility of the left mandibular premolar region. A poorly defined radiolucency is evident in the lesion and bone.	1. Dental abscess with osteomyelitis	Consultations: dentist, oral and maxillofacial surgeon (OMFS), pediatrician, infectious disease specialist
	2. Aggressive periapical cyst (apical periodontitis)	Laboratory and radiographic studies: blood tests: CBC, CRP, culture and sensitivity testing, panoramic X-ray and/or CT scan
	3. Juvenile ossifying fibroma	Treatment (medical and/or surgical): antibiotics
		Pain management: ibuprofen or acetaminophen
		Dental treatment: surgical intervention
		Additional considerations: supportive care (maintaining good oral hygiene, proper hydration, and a nutritious diet)
		Follow-up: regular monitoring
An 18-year-old male patient presented to the clinic with increasing pain in the right mandible for the past three months, with marked facial swelling and pain that was worse with activity and at night. There was no history of trauma or dental treatment in the affected area. On examination, there is a visible asymmetry of the right mandible with marked swelling extending from the right zygoma to below the lower mandibular border. Intraoral examination reveals a palpable hard mass in the right mandible, approximately 4×4 cm, palpable from the first molar to the anterior ramus border. The submandibular lymph nodes on the right are palpable, firm, and immobile. The lesion is tender for palpation. The panoramic radiograph shows a large, irregularly circumscribed, radiolucent lesion in the right mandibular body and ramus. Radiographically, the lesion has a mixed radiolucent/radiopaque appearance.	1. Aggressive odontogenic tumor (e.g., ameloblastoma)	Consults: oral and maxillofacial surgeon
	2. Osteomyelitis	Laboratory and radiographic studies: biopsy
	3. Mandibular malignancy	Imaging studies: X-ray and panoramic X-ray, CT scan
		Additional investigations: PET scans or MRI scans
		Treatments: marginal resection, segmental resection, mandibulectomy
		Additional considerations: reconstruction: bone grafting, dental implants, or prosthetics
		Adjuvant therapy: radiation therapy or chemotherapy
		Long-term follow-up: regular follow-up for any signs of recurrence and to manage potential side effects of treatment
A 42-year-old woman presented with a three-month history of painless pigmentation of the left upper anterior gingiva. There was no history of systemic disease or head and neck trauma. On examination, a palpable, tender, hard lymph node was found under the left mandible and a separate, painless, mobile lymph node on the right side. Intraoral examination revealed a non-tender, black pigmentation on the left maxillary gingiva extending to the adjacent teeth and palatal gingiva.	1. Melanoma	Consults: dermatologist, surgical oncologist, medical oncologist, radiation oncologist
	2. Amalgam tattoo	Laboratory and radiographic studies: biopsy, sentinel lymph node biopsy
	3. Pigmented nodular mucosal melanoma	Imaging studies: ultrasound, CT scan, PET scan
		Treatments (medical and/or surgical): wide excision, lymph node dissection
		Adjuvant therapy: immunotherapy, targeted therapy, radiation therapy
		Other considerations: clinical trials
		Supportive care: pain management, emotional support, and nutritional counseling

A total of 16 preliminary diagnoses from the prompts consisting of 12 clinical scenarios were common to both LLMs. It is also noteworthy that the most probable diagnosis (the diagnosis given in the first place) of seven prompts is common.

## Discussion

Oral and maxillofacial surgery has gradually evolved to include a wide range of pathological issues, including malignancies of the mouth, congenital deformities, and soft tissue and bone abnormalities. These pathologies sometimes overlap with other branches of medicine (such as otolaryngology, head and neck surgery, orthopedic surgery, and plastic surgery), which can complicate the consultation process, an important part of treatment and management [7]. Complications in the consultation process can potentially delay referral to an appropriate specialist and adversely affect survival. Clinicians tend to use protocols for identifying and treating lesions that they encounter more frequently in their daily routines. Major differences emerge between the interpretation of pathological formations encountered in clinical routine by human evaluators and evaluations by AI.

Differences in diagnosis between AI and humans

In their comprehensive review, Shen et al. [8] examined the disparities between the diagnostic capabilities of AI and human practitioners. That study revealed that current AI development exhibits diagnostic capabilities equal to or surpassing those of clinicians, particularly in image recognition tasks [8]. AI has achieved noteworthy milestones, with a primary focus on image recognition and the identification of objects in the realm of medical diagnoses [9]. Using computer-assisted technologies, AI rapidly detects clinical symptoms based on image attributes, thereby enhancing operational efficiency. AI particularly excels in diagnoses that rely on visual appearances, such as skin diseases, thus alleviating the cognitive burden on human experts [9]. AI exhibits particular proficiency in image analysis, especially in the context of disease diagnosis through image evaluation. Its intrinsic resilience to fatigue and continual learning capabilities contribute significantly to its success, even surpassing the abilities of clinical professionals in this specialized domain [10]. Nevertheless, AI's diagnostic accomplishments are dependent upon human clinicians who establish diagnostic criteria grounded in their own extensive practical experience. AI relies on human experts to guide its predictions, since it lacks the autonomy to independently interpret data or yield substantial and meaningful results [11]. The relationship between AI and human users underscores the indissoluble connection between advanced AI and its clinical relevance. While the development of AI suggests promising prospects for applications in the medical field, its efficacy hinges on evaluation by medical experts. In medical contexts, AI complements human expertise, enhancing the overall efficiency of clinical operations [10]. Nonetheless, challenges persist. The question of whether AI can completely replace assessments by clinicians remains unanswered, and it is conceivable that a hybrid system involving both AI and medical professionals may yield more effective diagnostic procedures [10]. Integrating AI into healthcare systems is of paramount importance due to its capacity to enhance precision and reduce time requirements in various aspects of the healthcare ecosystem [12]. The long-term advantages of incorporating AI across healthcare hold out the promise of heightened efficiency and precision within the sector. ChatGPT's potential applications in medicine span from identifying promising research areas to aiding professionals in clinical and laboratory diagnoses [12]. General-purpose AI models are increasingly facilitating the use of image-based AI in clinical imaging. Inception v3, a medical imaging model with cross-domain applicability, has emerged as a result. However, it is important to note that ChatGPT cannot generate images and can only provide verbal descriptions when prompted. ChatGPT's training data comprise general text content, and it may not therefore possess specialized medical knowledge or terminology. Its capacity to respond to specific medical queries or offer precise advice on medical matters may be somewhat limited in consequence [13].

Medical diagnosis and decision support

In the present study, ChatGPT accurately diagnosed and suggested reasonable treatments for clinical issues presented through medical scenarios with applicable jargon and relevant patient information. In alignment with findings from a similar study, inquiries formulated with appropriate medical terminology and comprehensive patient data elicited accurate diagnosis of clinical problems and reasonable treatment suggestions [14]. Providing a detailed diagnosis and treatment for common clinical problems presented by patients in non-medical jargon is likely to be perceived as an easy task by human evaluators but poses a challenge for AI systems. The gap between perceived difficulty and AI performance arises from the contrast between human and AI strengths and weaknesses. Humans, constrained by limited data storage, tend to rate rare conditions and treatments as more difficult, a limitation not applicable to AI [14]. Similar to the present study, Mago and Sharma [3] also employed a Likert scale to assess ChatGPT's performance in oral and maxillofacial radiology, oral pathologies, and anatomical landmarks. Those authors reported a remarkable accuracy rate of 100% for anatomical landmarks. However, it is also worth noting that their assessment of oral and maxillofacial pathologies was limited to major or characteristic radiographic features. In terms of mean scores for inquiries related to anatomical landmarks, oral and maxillofacial pathologies, and radiographic features of oral and maxillofacial pathologies, they determined mean Likert scale scores of 3.94, 3.85, and 3.96, respectively. It is also essential to emphasize that the median and mode scores across all categories were consistently 4, indicating a high level of agreement among the respondents [3]. The results therefore suggest that ChatGPT may represent a valuable tool for a wide range of oral pathologies, one on which experts in the field can confidently rely for assistance. At this stage, it will be useful to explore not only the diagnostic and treatment recommendation capabilities of ChatGPT but also its advanced use and advantages in that context.

Benefits of ChatGPT in scientific research

ChatGPT can represent a valuable resource for crafting a scientific paper's introductory paragraph. This initial section serves the crucial purpose of defining the research objectives and underlining the study's significance. ChatGPT can be of assistance in structuring and arranging the ideas the researcher wishes to communicate in the introductory paragraph, ultimately assisting in the creation of a clear and engaging thesis statement. This, in turn, will enhance the reader's comprehension of the study's purpose and its broader importance. ChatGPT can also play a supportive role in the writing process. Scientific writing typically entails the presentation of the research methodology and a description of the experiments conducted. ChatGPT can contribute to this process by helping researchers articulate these aspects in a clear and concise manner [15]. It can also offer suggestions for the selection of appropriate phrases and terminology that promote easy understanding, which is particularly advantageous for researchers whose native language is not English. Additionally, ChatGPT can be of assistance in sourcing relevant materials for the literature review section. It can also be of assistance in summarizing the essential findings of these sources. This dual function helps researchers streamline the focus of their work and present their findings with greater brevity and precision. An added benefit of incorporating ChatGPT into scientific writing is its capability to obviate plagiarism. ChatGPT is equipped with an anti-plagiarism feature that scrutinizes the written content against existing articles available online. This painstaking check helps identify potential instances of unintentional content duplication or plagiarism. By detecting these issues before the document is submitted for publication, ChatGPT can safeguard researchers from potential complications with publishing authorities [16].

ChatGPT in medical training and patient education

Together with other conversational agents, ChatGPT has the potential to revolutionize medical education and training. These tools provide personalized, interactive, and easily accessible learning experiences. By providing medical professionals with essential skills and knowledge, ChatGPT can play a role in enhancing patient outcomes and furthering progress in the field of medicine. Once its potential had been recognized, this tool underwent rigorous testing. In a recent study, ChatGPT demonstrated proficiency by passing all three sections of the United States Medical Licensing Exam (USMLE) [17]. Another study found that GPT-3.5 (consisting of Codex and InstructGPT) achieved human-level performance on various datasets, including USMLE (60.2%), MedMCQA (57.5%), and PubMedQA (78.2%)[18]. Balel [19] observed that ChatGPT provided reasonably precise and helpful responses to patient-centric inquiries within the realm of oral and maxillofacial surgery. They concluded that ChatGPT has significant potential as a patient education tool and is able to provide high-quality answers to patients' questions in pre- and postoperative care, particularly in cases of impacted teeth, dental implants, temporomandibular joint disorders, and orthognathic surgery [19]. One potential development might involve integrating ChatGPT with prominent databases such as Web of Science, PubMed, or Scopus, thereby further enhancing the quality and precision of responses to technical queries. Such an innovation would facilitate access on the part of academics and medical professionals to topic-specific information, ensuring they can obtain pertinent materials without the risk of overlooking critical literature, as is sometimes the case with conventional keyword-based searches [20]. 

Limitations of ChatGPT

While representing a remarkable language model, ChatGPT nevertheless has a number of limitations that require careful consideration. Hallucination is one particularly noteworthy concern [21]. The model might generate responses that seem plausible but lack a factual basis [22]. This challenge ties into its second limitation, its knowledge cut-off point in September 2021. This means that any information or developments after that date may be unknown to the model, leading to potentially inaccurate or outdated information [23]. Moreover, ChatGPT can be influenced by populism and political activism, reflecting biases in its training data and potentially echoing controversial perspectives, especially in political or social matters, thus raising concerns about its objectivity [24]. The trustworthiness of ChatGPT may be questionable in controversial situations, such as the COVID-19 pandemic [25]. The model might generate responses that align with popular beliefs or prevailing sentiments rather than relying on accurate and evidence-based information [25]. This has implications for its reliability as a source of information, especially in matters of public health and safety [26]. Inconsistency in responses poses another challenge. ChatGPT may offer differing responses to the same inquiry made at different times [23]. This variability may reduce the reliability and coherence of the information provided and create confusion for users seeking accurate and reliable answers. Finally, the risk of false references is another concern. ChatGPT may inadvertently cite non-existent sources or provide information that cannot be verified [23]. This poses challenges for users who rely on the model for accurate references and authoritative information. In navigating these limitations, it is essential for users to approach ChatGPT-generated content with a critical mindset, cross-referencing information where possible and recognizing the model's constraints in handling complex, dynamic, and controversial topics. Ethical concerns also arise when using ChatGPT, including potential issues of trustworthiness, infringement of intellectual property, copyright violations, and biases. It is crucial to conduct a thorough evaluation and address any limitations or ethical dilemmas before integrating ChatGPT into practice [27].

Strengths of the study

Another aim of this study was to evaluate ChatGPT's real-time assessments of clinical pathologies. The results were significantly better than anticipated, with diagnoses and appropriate treatment protocols found to be accurate in nearly all of the cases in all retrospectively designed scenarios. Correct case predictions were made in the interrogations conducted without using a magic word (e.g., the use of words such as "pregnant" and "trimester" in pregnancy tumors that might facilitate the identification of the phenomenon).

Shortcomings of the study

Assessment of identical ChatGPT outcomes by separate specialists (radiology and surgery), whereby surgeons evaluate treatment-related outputs and radiologists assess diagnosis-related outputs, could have a considerable impact on the statistics. In terms of obtaining more meaningful results, increasing the number and variety of samples (case variability) will permit the evaluation of all scores on the Likert scale, and it may be possible to submit all scores to statistical analysis. The importance of meticulous history-taking during questioning and clinical evaluation was highlighted in this study. However, some details may have been overlooked due to the retrospective study design. A prospective study might provide more valid results in the scenarios to be investigated.

Future recommendations

Better systems capable of detecting even small changes in data produced by ChatGPT now need to be developed. Evaluating ChatGPT's output currently requires careful examination by editors, and its difficulty in properly citing sources is a particular problem. However, ongoing research is addressing these issues and seeking solutions. Academic journals should consider setting clear rules about using AI in scholarly papers in order to prevent misuse [28]. Instead of merely looking for ChatGPT's weaknesses in order to identify academic misconduct, educators might usefully change assignment questions to focus on critical thinking skills, thus reducing the chances of misuse of technology [29]. In addition to ChatGPT, other chatbots with significant potential for use in the medical field are also available. For example, BioLinkBERT, DRAGON, Galactica, PubMed BERT, PubMed GPT, and Med-PALM 2 all demonstrate superior information generation capabilities in the medical field, but are currently affected by limitations in terms of accessibility [30].

## Conclusions

No previous studies have assessed pathology cases in the field of oral and maxillofacial surgery using ChatGPT. Although we were unable to achieve a sufficient number of cases at the time, our aim is to present a pioneering study by highlighting the advantages of AI in this context. Since this tool benefits from an easy-to-use interface, we hope to encourage further studies in order to assess ChatGPT's reliability in practical settings.
